# Telomere length, vitamin B12 and mortality in persons undergoing coronary angiography: the Ludwigshafen risk and cardiovascular health study

**DOI:** 10.18632/aging.102238

**Published:** 2019-09-06

**Authors:** Irene Pusceddu, Wolfgang Herrmann, Marcus E. Kleber, Hubert Scharnagl, Winfried März, Markus Herrmann

**Affiliations:** 1 Department of Clinical Pathology, Hospital of Bolzano, Bolzano, Italy; 2 Department of Clinical Chemistry, University of Saarland, Homburg, Germany; 3 Medical Clinic V - Nephrology, Hypertensiology, Rheumatology, Endocrinology, Diabetology, Medical Faculty of Mannheim, University of Heidelberg, Mannheim, Germany; 4 Medical University of Graz, Clinical Institute of Medical and Chemical Laboratory Diagnostics, Graz, Austria; 5 Synlab Academy, Synlab Holding Deutschland GmbH, Mannheim, Germany

**Keywords:** B12, telomere length, hyperhomocysteinemia, inflammation, mortality

## Abstract

Background: Vitamin B12 (B12) deficiency and excess are associated with increased risk of age-related-diseases and mortality. It has been suggested that high- and low-B12 concentrations link to increased mortality through accelerated genomic aging and inflammation. Evidence to support this is limited.

Results: B12 was associated with all-cause-mortality, RTL and hsCRP in a non-linear fashion. The association between B12 and mortality was not independent, as it lost significance after adjustment for potential confounders. In the lowest-(LB12) and highest-(HB12) quartiles of B12 mortality was higher than in the mid-range (HR:LB12:1.23;CI95%:1.06-1.43; HR:HB12:1.24;CI95%:1.06-1.44). We divided subjects with LB12 in quartiles of their RTL. Those with the longest-telomeres had a lower mortality-rate (HR:0.57;95%CI:0.39-0.83) and lower homocysteine than those with the shortest-telomeres. Amongst subjects with HB12, those with long-telomeres also had a lower mortality than those with short-telomeres (HR:0.40;95%CI:0.27-0.59). IL-6 and hsCRP concentrations were low in HB12LT but were high in HB12ST.

Methods: B12, homocysteine, telomere length (RTL), interleukin-6 (IL-6) and high-sensitive-C-reactive-protein (hsCRP) were measured in 2970 participants of the LURIC study.

Conclusions: Mortality, stratified according to B12 and RTL, seems to be driven by different mechanisms. In LB12 and HB12 subjects, mortality and accelerated telomere shortening might be driven by homocysteine and inflammation, respectively.

## INTRODUCTION

Short telomeres [1–3] and hyperhomocysteinemia (HHCY) [4, 5], induced by vitamin B12 (B12) deficiency, are associated with an increase mortality and occur commonly in elderly subjects [6]. B12 is an essential co-factor required for two enzymatic reactions in the human body. On one side, B12 is co-factor for the re-methylation of homocysteine (HCY) to methionine, a reaction catalyzed by the methionine synthase enzyme (MS) [7]. On the other side, B12 is co-factor for the isomerization of methylmalonyl CoA to succinyl CoA by the enzyme methylmalonyl CoA mutase [7]. In B12 deficiency, the activity of both enzymes is impaired leading to increased plasma concentrations of HCY and methylmalonic acid (MMA) [8]. Both these metabolic products are widely used as markers of B12 deficiency whereby compared to HCY, MMA, may be more specific and sensitive for B12 deficiency [8–10]. B12 has also been linked to genome stability [7]; it is necessary for the production of methyl groups required for DNA and histone methylation [11]. B12 is further required for the maintenance of an optimal anti-oxidative status [12]. Both these pathways, altered methylation and oxidative stress, induce cellular senescence, a condition also characterized by inflammation, altered cellular metabolism, genome instability and telomere dysfunction [13].

HHCY, induced by B12 deficiency, has been linked to mortality in several studies [4, 5, 14–16]. It has been hypothesized that HHCY triggers pro-oxidative reactions, which may cause increased mortality [15]. Surprisingly, it has been shown that B12 overload is also associated with increased all-cause mortality in several study cohorts [17–20], but this association disappeared after adjustment for liver biomarkers [20]. It has been speculated that liver damage can induce high B12 levels, by mechanisms including hepatocyte lysis with subsequent release of stored B12 in the form of holohaptocorrin, decreased liver production of transcobalamin II with consequent decreased B12 uptake, and decreased liver uptake of holotranscobalamin with consecutively increased amounts of circulating B12 [20].

Increased mortality has also been reported in subjects with telomere dysfunction [1–3]. Telomeres are the ends of chromosomes and protect them from degradation and unwanted recombination and are involved in the maintenance of genomic stability [13]. Recently, we reported that participants of the Ludwigshafen Risk and Cardiovascular Health (LURIC) study with the longest telomeres (4^th^ quartile) had a markedly lower hazard ratio (HR) for all-cause (0.42) and CVD (0.40) mortality than those with the shortest telomeres (1^st^ quartile) [3].

Until today, the relationship between telomere length and B12 has not been studied comprehensively. The very few existing studies reported no significant association [21–24]. However, these studies are limited by a relatively small sample size and the lack of mortality data. Therefore, we analyzed the relationship between B12 status, mortality and telomere length in the LURIC study, a large prospective observation study with a median follow-up of 9.9 years.

## RESULTS

### Characteristics of the study population

The general characteristics of the study population were previously reported [25]. Briefly, the median age of the cohort was 63.5 years. The median B12 concentration was 344 pmol/L. Table 1 shows major cardiovascular risk factors, medications, B vitamin status and markers of inflammation in the entire cohort and according to B12 status (1^st^ quartile, mid-range and 4^th^ quartile, n=3312). During a median follow-up period of 9.9 years 2321 patients stayed alive and 995 died. Of the 2970 participants in whom the measurements of B12 and RTL were complete, 2067 patients stayed alive and 903 died.

**Table 1:** Characteristics of the entire cohort and according to low, mid-range and high B12.

**Parameters**	Enthire cohort n=3316*	1° quartile B12 (< 259 pmol/L) n=834*	Mid-range B12 (260-472 pmol/L) n=1650*	4° quartile B12 (>473 pmol/L) n=828*	**p-Value mid-range vs 1° quartile**	**p-Value mid-range vs 4° quartile**
Sex (M, %)	69.5	70.7	70.8	65.9	0.646	**0.008**
Age (years)	63.5 (48.1-75.6)	65.3 (48.7-77.1)	62.9 (47.9-74.9)	62.8 (47.4-75.3)	**<0.001**	0.990
Death (%)	30.0	29.9	27.3	32.2	**0.006**	**0.013**
*Cardiac causes (%)*	*57*	*54*	*58*	*60*	0.286	**0.045**
*Fatal stroke (%)*	*6*	*9*	*5*	*6*	**0.025**	0.281
*Fatal infection (%)*	*8*	*7*	*8*	*9*	0.873	0.281
*Fatal cancer (%)*	*15*	*15*	*17*	*9*	0.784	**0.037**
*Other causes (%)*	*14*	*15*	*12*	*16*	0.077	0.054
Diabetes mellitus (%)	39.8	38.6	38.9	42.8	0.706	0.075
CAD (%)	77.8	79.7	77.1	77.1	0.322	0.848
Previous MI (%)	41.2	40.4	41.5	41.5	0.503	0.822
Previous stroke (%)	9.1	11.2	7.8	6.9	**0.007**	0.133
PVD (%)	9.6	11.7	8.9	8.8	**0.036**	0.900
Acute Infection (%)	9.6	7.6	9.9	10.8	**0.044**	0.511
SBP (mmHg)	140 (111-173)	142 (112-173)	141 (112-173)	138 (109-172)	**0.007**	**0.024**
DBP (mmHg)	81 (66-96)	80 (67-97)	81 (67-97)	80 (65-95)	**0.003**	**0.001**
BMI (kg/m^2^)	27.1 (22.9-32.7)	27.2 (22.8-33.2)	27.2 (22.9-32.9)	26.8 (22.6-32.4)	**0.004**	**0.002**
ACE-inhibitors (%)	53.3	13.5	25.9	13.9	0.387	0.094
Any lipid lowering therapy (%)	48.4	11.9	24.5	12	0.325	0.591
CSE inhibitors (statins) (%)	46.9	11.5	23.8	11.5	0.275	0.412
Non-statin lipid-lowering drugs (%)	2.4	0.6	1.1	0.7	0.955	0.414
Aspirin/other antiplatelet agents (%)	71.4	18.2	35.7	17.4	0.849	0.297
Beta blockers (%)	63.3	15.7	32	15.5	0.419	0.265
Vitamin K antagonists (%)	6.7	1.5	3.3	1.9	0.638	0.414
Diuretics (%)	28.6	6.8	13.8	8	0.658	**0.026**
Thyroid therapy (%)	10.1	2.5	5.1	2.6	0.785	0.948
Vitamin supplementation (%)	2.4	0.5	1.1	0.9	0.525	0.052
Hemoglobin (g/dL)	13.9 (11.9-15.6)	13.7 (11.7-15.5)	14.0 (12.2-15.7)	13.8 (11.7-15.7)	**<0.001**	**0.004**
HbA1c (%)	6.0 (5.2-7.9)	6.0 (5.2-7.4)	6.0 (5.3-7.9)	6.1 (5.2-8.2)	**0.014**	0.143
LDL cholesterol (mg/dL)	114 (75-159)	116 (76-159)	115 (76-160)	112 (74-157)	0.167	0.082
HDL cholesterol (mg/dL)	37 (26-53)	37 (26-54)	38 (27-53)	37 (26-53)	0.307	0.279
TSH (mU/L)	1.24 (0.38-2.86)	1.21 (0.4-2.94)	1.21 (0.36-2.75)	1.33 (0.4-3.06)	0.747	**0.003**
B12 (pmol/L)	344 (197-633)	210 (145-250)	344 (278-435)	589 (489-945)	-	-
Vitamin B6 (μg/L)	8.9 (3.5-22.1)	7.4 (3.1-17.1)	9.2 (3.5-20.6)	10.1 (3.78-29.7)	**<0.001**	**<0.001**
Folate (μg/L)	7.9 (4.7-12.1)	7.0 (4.3-11.2)	7.8 (4.8-11.9)	9 (5.1-13.2)	**<0.001**	**<0.001**
HCY (μmol/L)	12.4 (8.1-19.8)	13.8 (8.8-22.97)	12.1 (8.3-18.7)	11.3 (7.3-18.9)	**<0.001**	**<0.001**
RTL	1.7881 (0.4651-4.9341)	1.8723 (0.4798-5.1521)	1.7048 (0.4321-4.9260)	1.9474 (0.5287-4.8783)	0.088	**0.008**
Age-corrected RTL	0.0280 (0.0070-0.0865)	0.0289 (0.0073-0.0848)	0.0267 (0.0063-0.870)	0.0315 (0.0086-0.00870)	0.337	**0.009**
hsCRP (mg/L)	3.39 (0.66-21.80)	3.34 (1.21-11.14)	2.97 (0.67-19.30)	3.91 (0.74-23.40)	**0.001**	**<0.001**
IL-6 (pg/mL)	3.20 (1.17-11.19)	3.67 (0.62-26.75)	3.04 (1.17-10.83)	3.33 (1.14-12.58)	**0.033**	**0.017**

CAD: coronary artery disease, MI: myocardial infarction, PVD: peripheral vascular disease, SBP: systolic blood pressure, DBP: diastolic blood pressure, BMI: body mass index, ACE: angiotensin converting enzyme, CSE: cholesterol synthesis enzyme, HbA1c: glycated hemoglobin, LDL: low density lipoprotein, HDL: high density lipoprotein, TSH: thyroid stimulating hormone, RTL: relative telomere length, hsCRP: high sensitive C-reactive protein, IL-6: interleukin 6.

*The entire cohort is composed by 3316 subjects. B12 was analyzed in 3312. The number of subjects of each quartile refers to those with a measurement of B12.

Statistically significant differences are reported in bold.

### B12 and mortality

**Figure 1:**
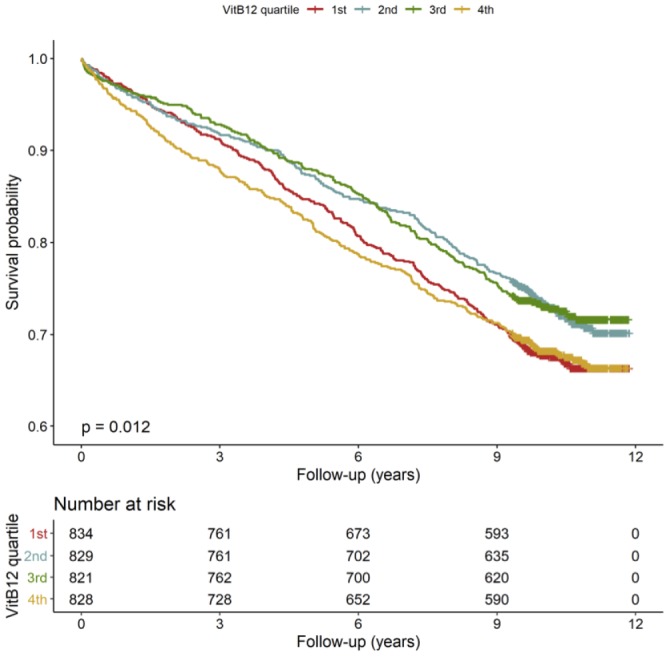
**Kaplan-Meier plots.** Cumulative survival according to quartiles of B12 (n=3312).

The mortality rates differed according to B12 quartiles. The Kaplan–Meier curves showed increased mortality rates in the 1^st^ and 4^th^ quartile of B12 (Figure 1, n=3312). Subjects in the 1^st^ (<259 pmol/L, n=834) quartile of B12 were characterized by a 12.3 % higher risk to die during follow-up compared to those in the mid-range of B12 (260-472 pmol/L, n=1650) (Table 2 Model 1). Similar results were obtained in the model adjusted for major cardiovascular risk factors, comorbidities, severity of CAD, medications and vitamin supplementation (Table 2 Model 2). However, these results changed substantially with further adjustment for age-corrected RTL (Table 2 Model 3), vitamin B6, folate, HCY, markers of inflammation like IL-6 and hsCRP, and markers of liver dysfunction, like albumin, GOT, GPT, GGT and total bilirubin (Table 2 Model 4).

**Table 2:** Risk for all-cause mortality according to B12 quartiles (n=3312).

**B12 quartiles (pmol/L)**	**No. of deceased (%) / alive (%) patients**	**Model 1 HR (95%CI)**	**P**	**Model 2 HR (95%CI)**	**P**	**Model 3 HR (95%CI)**	**P**	**Model 4 HR (95%CI)**	**P**
Mid-range (260-472) (n=1650)	453 (27%) / 1197 (73%)	Ref.		Ref.		Ref.		Ref.	
1^st^ (<259) (n=834)	273 (33%) / 561 (67%)	1.23 (1.06-1.43)	**0.007**	1.19 (1.02-1.39)	**0.024**	1.16 (0.99-1.36)	0.071	1.05 (0.88-1.25)	0.592
4^th^ (>473) (n=828)	267 (32%) / 561 (68%)	1.24 (1.06-1.44)	**0.006**	1.15 (0.99-1.34)	0.074	1.13 (0.96-1.32)	0.148	1.10 (0.92-1.32)	0.278

Model 1: crude model. Model 2: adjusted for sex, LDL-C, HDL-C, BMI, blood pressure, diabetes mellitus, smoking, CAD, MI, stroke, PVD, acute infection, TSH, creatinine, drugs (ACE-inhibitors, lipid lowering therapy, aspirin/other antiplatelet agents, beta blockers, vitamin K antagonist, diuretics, thyroid therapy, vitamin supplementation, Friesinger Score, Gensini Score. Model 3: as model 2 plus age-corrected RTL; Model 4: as 3 plus vitamin B6, folate, HCY, IL-6, hsCRP, albumin, total bilirubin, GOT, GPT, GGT, ALP. Ref.: reference. Significant in bold.

Subjects in the 4^th^ (>473 pmol/L, n=828) quartile of B12 plasma concentration were characterized by a 12.4 % higher risk to die during follow-up compared to those in the mid-range of B12 (260-472 pmol/L, n=1650) (Table 2 Model 1). However, these results changed substantially with further adjustment for major cardiovascular risk factors, comorbidities, severity of CAD, medications and vitamin supplementation (Table 2 Model 2), age-corrected RTL (Table 2 Model 3), vitamin B6, folate, HCY, IL-6, hsCRP, albumin, GOT, GPT, GGT and total bilirubin (Table 2 Model 4).

### B12, mortality and age-corrected RTL

To further analyze the association of subjects in the 1^st^ and 4^th^ quartile of B12 with higher mortality, we analyzed age-corrected RTL, another import biomarker of all-cause mortality [3]. The statistical analyses were performed only in subjects in whom the measurements of B12 and RTL were complete (n=2970).

**Figure 2:**
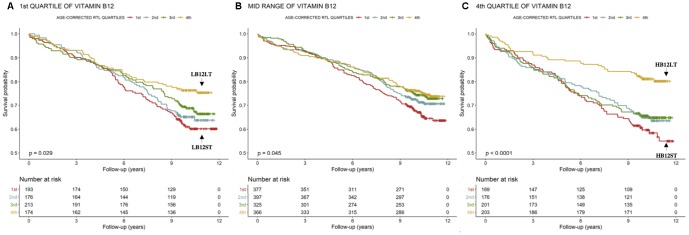
**Kaplan-Meier plots.** Cumulative survival according to age-corrected RTL within the 1^st^, mid-range and 4^th^ quartiles of B12.

LB12LT (low B12 and long telomeres, n=174) subjects had a lower mortality-rate compared to all the other subjects (Figure 2A). The Cox-regression analysis revealed that LB12LT patients were characterized by a 43% reduced risk to die during follow-up in the unadjusted model compared to those in the 1^st^ age-corrected RTL quartile (LB12ST, n=193, Table 3A Model 1). In order to include other confounding factors in the Cox-regression, we applied different models: LB12LT patients were also characterized by a 43% reduced risk to die during follow up in a model adjusted for markers of inflammation, liver function and B vitamin status compared to LB12ST (n=193, Table 3A Model 2). However, the additional adjustment for major cardiovascular risk factors, CAD severity, medications and vitamin supplementation disrupted this relationship (Table 3A Model 3).

**Table 3:** Risk for all-cause mortality according to age-corrected RTL quartiles in B12 low, mid-range and high (n=2970, only subjects in whom the measurements of B12 and RTL were complete).

**(A) 1^st^ quartile of B12 (n=756)**							
**Age-corrected RTL quartiles**	**Nr. of deceased(%) / alive (%) patients**	**Model 1 HR (95%CI)**	**P**	**Model 2 HR (95%CI)**	**P**	**Model 3 HR (95%CI)**	**P**
1^st^ (<0.0140) (LB12ST) (n=193)	75 (39%) / 118 (61%)	Ref.		Ref.		Ref.	
2^nd^ - 3^rd^(0.0141-0.0509) (n=389)	131 (34%) / 258 (66%)	0.84 (0.63-1.12)	0.232	0.78 (0.56-1.08)	0.137	0.77 (0.54-1.09)	0.137
4^th^ (>0.0510) (LB12LT) (n=174)	42 (24%) / 132 (76%)	0.57 (0.39-0.83)	**0.004**	0.57 (0.37-0.87)	**0.009**	0.75 (0.48-1.17)	0.211
**(B) Mid-range B12 (n=1465)**							
**Age-corrected RTL quartiles**		**Model 1 HR (95%CI)**	**P**	**Model 2 HR (95%CI)**	**P**	**Model 3 HR (95%CI)**	**P**
1^st^ (<0.0140) (n=377)	126 (33%) / 251 (67%)	Ref.		Ref.		Ref.	
2^nd^ - 3^rd^(0.0141-0.0509) (n=722)	196 (27%) / 526 (73%)	0.79 (0.63-0.99)	**0.037**	0.84 (0.65-1.08)	0.169	0.78 (0.60-1.01)	0.061
4^th^ (>0.0510) (n=366)	92 (25%) / 274 (75%)	0.70 (0.54-0.92)	**0.011**	0.80 (0.59-1.09)	0.156	0.82 (0.60-1.13)	0.223
**(C) 4^th^ quartile of B12 (n=749)**							
**Age-corrected RTL quartiles**		**Model 1 HR (95%CI)**	**P**	**Model 2 HR (95%CI)**	**P**	**Model 3 HR (95%CI)**	**P**
1^st^ (<0.0140) (HB12ST) (n=169)	70 (41%) / 99 (59%)	Ref.		Ref.		Ref.	
2^nd^ - 3^rd^(0.0141-0.0509) (n=377)	132 (35%) / 245 (65%)	0.83 (0.62-1.11)	0.209	0.86 (0.61-1.20)	0.368	0.87 (0.60-1.24)	0.434
4^th^ (>0.0510) (HB12LT) (n=203)	39 (19%) / 164 (81%)	0.40 (0.27-0.59)	**<0.001**	0.51 (0.33-0.79)	**0.003**	0.62 (0.39-0.98)	**0.040**

Model 1: crude model. Model 2: adjusted for IL-6, hsCRP, albumin, total bilirubin, GOT, GPT, GGT, ALP, HCY, vitamin B6 and folate. Model 3: as model 2 plus sex, LDL-C, HDL-C, BMI, blood pressure, diabetes mellitus, smoking, CAD, MI, stroke, PVD, acute infection, creatinine, TSH, ace-inhibitor, lipid lowering therapy, aspirin/other antiplatelet agents, beta blockers, vitamin K antagonist, diuretics, thyroid therapy, vitamin supplementation, Friesinger Score, Gensini. Score. Ref.: reference.

Statistically significant differences are reported in bold.

Further, the LB12LT (n=174) subjects were characterized by significantly lower concentrations of HCY compared to all the other subjects (Table 4A).

**Table 4:** Median (10^th^ - 90^th^ percentiles) values for HCY, vitamin B6, folate, hsCRP and IL-6 according to quartiles of age-corrected RTL in 1^st^ (A), mid-range (2^nd^-3^rd^) and 4^th^ (C) quartiles of B12 (n=2970, only subjects in whom the measurements of B12 and RTL were complete).

**(A) 1^st^ quartile of B12**			
	**Age-corrected RTL quartiles**		
	**1, 2, 3** (n=582)	**4^th^** (n=174) (LB12LT)	**p-Value**
HCY (μmol/L)	14.2 (9.1-23.8)	12.8 (8.3-22.4)	**<0.001**
Vitamin B6 (μg/L)	7.3 (3.2-16.6)	8.1 (3.2-17.5)	0.237
Folate (μg/L)	6.7 (4.2-11.1)	7.1 (4.7-11.3)	0.152
hsCRP (mg/L)	3.40 (0.62-23.80)	4.01 (0.52-26.25)	0.458
IL-6 (pg/mL)	3.34 (1.21-11.51)	3.39 (1.29-10.73)	0.927
**(B) Mid-range (2^nd^ -3^rd^ quartiles) of B12**			
	**Age-corrected RTL quartiles**		
	**1, 2, 3** (n=1099)	**4^th^** (n=366)	**p-Value**
HCY (μmol/L)	12.2 (8.4-18.8)	11.8 (7.9-18.6)	0.071
Vitamin B6 (μg/L)	8.9 (3.5-20.4)	10.2 (3.7-22.4)	**0.006**
Folate (μg/L)	7.6 (4.7-11.9)	8.1 (4.9-11.6)	0.382
hsCRP (mg/L)	3.16 (0.68-20.26)	2.62 (0.62-17.55)	0.055
IL-6 (pg/mL)	3.20 (1.21-11.18)	2.73 (1.14-9.14)	**0.015**
**(C) 4^th^ quartile of B12**			
	**Age-corrected RTL quartiles**		
	**1, 2, 3** (n=546)	**4^th^** (n=203) (HB12LT)	**p-Value**
HCY (μmol/L)	11.3 (7.2-20.1)	10.7 (7.5-17.4)	0.125
Vitamin B6 (μg/L)	9.8 (3.7-30.6)	11.1 (3.9-29.7)	0.274
Folate (μg/L)	8.8 (5.1-12.8)	9.1 (5.0-12.9)	0.555
hsCRP (mg/L)	3.91 (0.88-26.15)	3.67 (0.43-21.64)	**0.006**
IL-6 (pg/mL)	3.78 (1.28-13.1)	2.62 (0.88-9.56)	**<0.001**

Statistically significant differences are reported in bold.

HB12LT subjects (high B12 and long telomeres, n=203) were characterized by a significantly lower mortality-rate compared to all the others (Figure 2C). In addition, the HB12LT subjects (n=203) were characterized by a 60% reduced risk to die during follow up in the unadjusted model compared to HB12ST (n=169, Table 3C Model 1). Further adjustment with markers of inflammation, liver function, B vitamin status (Table 3C Model 2), major cardiovascular risk factors, CAD severity, medications and vitamin supplementation (Table 3 Model 3) did not significantly change these results.

Finally, the HB12LT subjects (n=203) were characterized by significantly lower concentrations of markers of inflammation like IL-6 and hsCRP (Table 4C). The Cox-regression analysis and the vitamin B-inflammatory status of subjects in the mid-range (2^nd^ and 3^rd^ quartile) of B12 and are reported in Table 3B, and Table 4B, respectively.

Finally, subjects in the 1^st^ and 4^th^ quartiles of B12 and with age-corrected RTL in the lower three quartiles had higher mortality rate (HR:1.56, 95%CI:1.12-2.17, p<0.001; HR:2.18, 95%CI:1.55-3.07, p=0.009, respectively), compared to those in the 4^th^ quartile of age-corrected RTL.

### B12, age-corrected RTL and markers of inflammation

**Figure 3:**
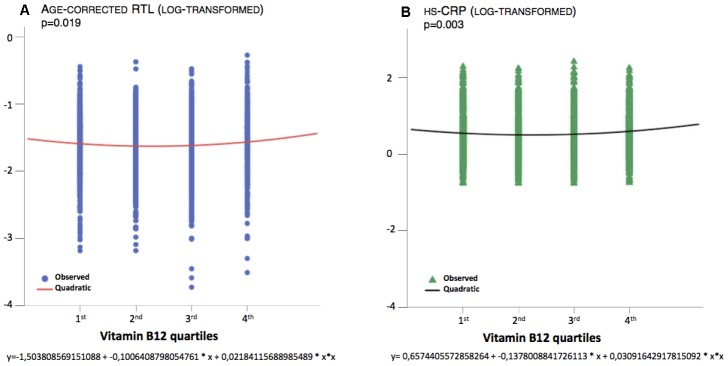
**Quadratic association between B12 and age-corrected RTL and hsCRP.**

The correlation between B12 and age-corrected RTL was non-linear, as shown in Figure 3A. The equations that describe the relationships between B12 and age-corrected RTL (Figure 3A, p=0.019) and between B12 and uncorrected RTL (p=0.010) are quadratic. Indeed, age-corrected RTL and uncorrected RTL are longer in subjects in the 1^st^ quartile of B12 concentration compared to those in the mid-range, although the difference was not statistically significant (Table 1). Age-corrected RTL and uncorrected RTL are also longer in subjects in the 4^th^ quartile of B12 concentration compared to those in the mid-range (p=0.009, p=0.008, respectively, Table 1). A similar non-linear relationship was also present for B12 and hsCRP, as shown in Figure 3B and Table 1.

## DISCUSSION

The present study provides clear evidence for a non-linear association between B12, telomere length and inflammation with mortality. In particular, subjects with B12 deficiency as well as B12 excess were characterized by a higher mortality rate compared to those with B12 in the mid-range. Our results also support previous studies that identified RTL as a strong predictor of mortality [1–3]. Both parameters, B12 and RTL, appear to be differentially regulated and a clear link between these two seems to be missing. Possibly, a dysregulated methyl group metabolism could be a link between vitamin B12 and RTL. In an earlier prospective B vitamins supplementation study, we suggested a possible effect of B vitamins on telomere biology in blood cells [26]. In another pilot study we suggested a functional relationship between one-carbon metabolism and telomere length [27]. Suboptimal B vitamins status and hyperhomocysteinemia are associated with altered DNA methylation and telomere length. The availability of nucleotides and methylation groups seems to impact telomere length. Additionally, sub-group analyses point towards telomere length as a potential common mediator of mortality risk in individuals with high and low B12 concentrations. This could imply that low or high B12 concentrations alone are not sufficient to increase mortality risk. Only if this occurs in conjunction with short telomeres mortality risk rises. Our results further suggest that the mechanisms causing short telomeres in subjects with high and low B12 concentrations are not the same. In B12 deficient individuals HCY appears to be a potential causative factor whereas chronic systemic inflammation might promote telomere shortening and mortality in subjects with B12 excess.

### B12 deficiency and mortality

B12 deficiency is common in elderly subjects [6], in hospitalized patients [28], and in ill patients [29]. The main causes of B12 deficiency are a reduced dietary intake and malabsorption [7]. In addition, aging and frailty contribute to intrinsic B12 deficiencies independently of nutritional intake [30]. B12 deficiency is linked with an increased risk to develop age-related diseases such as neurodegenerative and cardiovascular disease as well as osteoporosis [6, 31]. Despite this increased risk of age-related diseases, a higher mortality in B12 deficient individuals has not been reported so far.

Here we show that patients in the 1^st^ quartile of B12 have a higher mortality compared to those in the mid-range of B12, in the crude model and after adjustment for common confounders. Amongst individuals with low B12, those with longer telomeres (LB12LT) have a lower average HCY concentration and a reduced mortality compared to those with the shortest telomeres (LB12ST). Both, HHCY [4, 15, 16] and shorter telomere length [1–3] are established predictors of all-cause and cardiovascular mortality, but have not been analyzed simultaneously in the same cohort. HCY is a cytotoxic metabolite of the methionine cycle that is located at the crossroad between two metabolic pathways. In the transsulfuration pathway HCY is detoxified to glutathione, the main anti-oxidant compound. In the re-methylation cycle, HCY is converted to S-adenosyl-methionine (SAM), the universal methyl-group donor required for virtually all methylation reactions [7]. Therefore, HCY is an important modulator of the cell’s methylation capacity and oxidative status. A pro-oxidative environment and a limited cellular methylation capacity promote DNA damage in subjects with HHCY. We speculate that B12 deficiency increases mortality through increased HCY, which causes oxidative DNA damage, DNA hypomethylation and accelerated telomere shortening.

In addition to the putative genomic effects of HCY, there are several other aspects that may contribute to cardiovascular mortality and the occurrence of cardiac arrhythmias [32, 33]. For example, high HCY can favor the occurrence of ischemic stroke and other fatal cardiovascular events through direct effects on atrial ion channels that trigger cardiac arrhythmias, such as atrial fibrillation [34, 35]. Moreover, it has been suggested that systemic inflammation promotes the incidence of cardiac arrhythmias through direct and indirect effects on the electric stability of the myocardium [36]. Inflammatory cytokines, particularly IL-6, do also seem to modulate the expression and function of specific ion channels in the cardiomyocyte [37].

### B12 excess and mortality

The present results show an increased mortality not only in the 1^st^ quartile of B12, but also in the 4^th^ quartile. Although high B12 has been linked to an increased mortality in previous studies [17–20] this association is not widely known. For example, in 2239 critically ill patients, elevated B12 levels (>1593 pmol/L) were associated with an increased 90-day mortality [17]. In the Newcastle 85+ study, subjects with B12 >500 pmol/L had a 40% higher risk of all-cause mortality compared to those with B12 <500 pmol/L (HR:1.41, 95%CI: 1.02-1.95, p=0.039) [18]. In another study of 129 heart failure patients and 50 healthy controls, B12 concentrations above 270 pg/mL had 80% sensitivity and 58% specificity for predicting all-cause mortality (area under the ROC curve = 0.67, 95%CI 0.56-0.78; p=0.003) [15]. A large observational study by Callaghan et al. showed unadjusted odds ratios of 2.83 (95%CI 2.13-3.76) and 2.72 (95%CI 2.08-3.55) for 30-days and 90-days death, respectively, in subjects with B12 concentration >1000 pmol/ [20]. Adjustment for demographic variables (age, gender, race and insurance type) and other common confounders did not change these results [20]. However, after further adjustment for liver function (albumin, GOT, GPT, ALP, total protein and total bilirubin) mortality did no longer differ between quartiles of B12 [20]. This suggests that liver dysfunction may mediate the increased mortality in subjects with high B12. The liver is directly involved in B12 metabolism through the uptake of B12 from the circulation, storage of B12 inside the hepatocytes and production of transcobalamin II [20]. Therefore, liver damage can contribute to excessive B12 levels through a reduced uptake from the circulation, release of intracellular B12 stores from damaged hepatocytes and a reduced production of transcobalamin II of B12 [20]. Also, in the present study high B12 was no longer predictive of all-cause mortality after adjustment for liver function tests. In summary these results suggest that it is the disturbed liver function itself rather than an excessive B12 plasma concentration that drives mortality.

At this point it should also be mentioned that not all previous studies were able to show an increased mortality in individuals with high B12 concentrations [38–40]. Differences in study design and variable B12 cut-offs may explain this discrepancy. The fact that holotranscobalamin, the metabolically active form of B12, accounts for only 10 to 30 % of all circulating B12 is another limiting factor. Holohaptocorrin the dominant form of B12, is metabolically inactive and stored in large amounts in the liver [8].

Another important observation in the present study was that high B12 also lost its predictive power after adjustment for RTL and inflammatory markers. Patients with high B12 and long telomeres (HB12LT) were characterized by a lower mortality rate and by lower concentrations of hsCRP and IL-6. Based on these results it can be speculated that in subjects with high B12 concentrations systemic inflammation increases mortality risk through accelerated telomere shortening. This concept is supported by a previous study in which we showed a negative correlation between RTL and markers of inflammation [14]. Other studies that analyzed the association between B12 and systemic inflammation, reported conflicting results. While some studies found significant associations, others did not [41–44]. For example, in 154 patients with type 2 diabetes mellitus Lee et al. reported an inverse correlation between B12 and IL-6 [43]. In contrast, treatment of 150 women with a combination of folic acid, vitamin B6 and B12 for an average of 7.3 years had no effect on hsCRP, IL-6 and markers of endothelial dysfunction [44]. In line with this result, HCY-lowering through B-vitamin supplementation has also failed to lower inflammatory plasma markers [44–46].

### B12 and telomere length

Only few studies analyzed the relationship between B12 and RTL [21–24]. None of these studies identified an association between RTL and B12 [21–24]. Only in one cohort study of 798 subjects, those with hsCRP >2 mg/L, showed an inverse correlation between B12 and RTL [24]. In contrasts to all previous studies that performed linear analyses, we show a non-linear, U-shaped association between B12 and RTL. This finding is intriguing because in previous studies others and we have demonstrated an inverse linear relationship between RTL and mortality [1–3]. Although the underlying mechanism of this non-linear relationship remains insufficiently understood, our results point towards telomere length rather than B12 as the principal driver of mortality. This hypothesis is supported by the fact that amongst individuals within the 1^st^ and 4^th^ quartile of B12, respectively, mortality was highest in those with the shortest telomeres. In a previous analysis of the LURIC study we could show that participants with the longest telomeres had a 42% lower risk to die compared to those with shorter telomeres [3]. As mentioned before, the mechanisms causing short telomeres in subjects with high and low B12 concentrations may not be the same. In B12 deficient individuals, HCY appears to be potentially causative whereas chronic systemic inflammation might promote telomere shortening and mortality in subjects with B12 excess. Both factors, HHCY and chronic systemic inflammation have repeatedly been shown to increase mortality [4, 14, 47, 48].

Another open question is why average RTL is longer amongst individuals within the 1^st^ and 4^th^ quartile of B12? It can be speculated that accelerated telomere shortening in B12 deficient subjects might be counteracted by compensatory mechanism that activate telomerase, an enzyme that can prolong telomeres. In oncologic patients, the induction of telomerase is a common tumor escape mechanism [49]. In contrast, average RTL in individuals with high B12 is probably the result of an adequately functioning one-carbon metabolism. Based on previous reports [20] it can be hypothesized that the higher mortality in these patients is cause by an independent systemic inflammatory disease that releases intracellular B12 from tissue stores. Although this theory seem appealing, robust scientific evidence is currently lacking.

### Limitations and other considerations

As B12 is an essential nutrient information about diet, calorie intake and vitamin supplementation could be of interest in this study. Only 81 subjects of the 3316 participants of the LURIC study were supplemented with vitamins. Because of such a small number we did not consider this group separately for statistical evaluation. Furthermore, detailed dietary information was not available.

It should be mentioned that total B12 is not the best surrogate marker for assessing the B12 status [8, 27]. The functional markers HCY and MMA, or holotranscobalamin are more informative [8, 27].

It also has to be acknowledged that extrapolating the impact of a single factor, such as B12, in a heterogeneous cohort of patients in which some have received polytherapy harbors substantial limitations that needs to be considered when interpreting the present results.

Finally, this examination has been conducted in Caucasian persons scheduled for coronary angiography who are at intermediate to high risk of dying. Our conclusions therefore must not unconditionally be extrapolated to other ethnicities of persons with different baseline characteristics.

### Concluding remarks

Extreme B12 concentrations are associated with increased mortality risk. Amongst individuals with low or high B12 those with the shortest telomeres have the highest risk to die. Accelerated telomere shortening might be a common promotor of mortality in these individuals. Our results further suggest that B12 and RTL are differentially regulated, and the mechanisms causing short telomeres in subjects with extreme B12 concentrations are not the same. While HHCY might drive telomere shortening in the context of B12 deficiency, systemic inflammation is a potential accelerator of telomere attrition in individuals with high B12. In view of the limitations associated with the measurement of B12 and RTL, additional markers, such as HCY, IL6 or hsCRP, should be considered when assessing patient’s mortality risk.

## MATERIALS AND METHODS

### Study design

We analyzed baseline blood samples and clinical outcome data from 2970 Caucasian participants of the LURIC study (n=3316) in whom the measurements of B12 and RTL were complete. A detailed description of the LURIC study has been published [25]. Briefly, patients hospitalized between June 1997 and January 2000 for elective diagnostic coronary angiography at the Heart Center Ludwigshafen (Germany) were enrolled. Inclusion criteria were: German ancestry, clinical stability except for acute coronary syndromes and the availability of a coronary angiogram [25]. Exclusion criteria were: any acute illness other than acute coronary syndromes. Consequently, subjects with decompensated heart failure, decompensated valvular disease, major infections, endocrine disease, or any type of surgery within the previous three months were excluded [25]. Furthermore, we excluded chronic multimorbid patients with predominant non-cardiac diseases, such as severe rheumatoid arthritis, persistent incapacitation after accident/trauma, and with malignancies within the past five years [25]. All patients underwent physical examination, coronary angiography, electrocardiography and blood collection [25]. A standardized questionnaire was used to record the medication history including the brand name, dose, duration, and time of last intake (in reference to the time of blood sampling) of any medication taken within the previous four weeks [25]. Medications included: ACE inhibitors, aspirin/other antiplatelet agents, beta blockers, vitamin K antagonists, diuretics, lipid lowering therapy (including statins and non-statin lipid-lowering drugs), thyroid therapy and vitamin supplementation. The severity of coronary artery disease (CAD) was quantified with the Friesinger Score [50] and the Gensini Score [51].

Median follow-up time was 9.9 years. Information about survival was obtained from local person registries. Two physicians blinded to participant’s baseline characteristics classified causes of death by reviewing hospital records and death certificates. In the case of disagreement about classification, the final decision was made by one of the principal investigators of LURIC study (W.M) after appropriate review of the data. The study was approved by the ethics committee of the Physicians Chamber of Rheinland-Pfalz and performed in accordance with the declaration of Helsinki [25]. All participants gave written informed consent [25].

### Laboratory analyses

B12 (n=3312) and folate (n=3315) were measured with automated immunoassays on an AXSYM analyzer (Abbott, USA) [52], high-sensitive C-reactive protein (hsCRP, n=3310) by immunonephelometry (Nephelometer II, Dade Behring, Germany) and IL-6 (n=3306) by ELISA (R&D Systems Inc. USA) [51]. Total HCY (n=3312) was measured by high performance liquid chromatography (HPLC; Waters, USA) [53]. Vitamin B_6_ (n=3311) was measured by HPLC (Waters Millennium chromatography with fluorescence detector 470, Immundiagnostik GmbH, Bensheim, Germany).

RTL (n=2974) was measured in genomic DNA using a quantitative-polymerase chain reaction (Q-PCR)-based assay, as previously reported [3]. Briefly, in each run 40 ng of sample DNA was analyzed in duplicate, a coefficient of variation (CV) between replicates of 2.5% was considered acceptable and the average of both replicates was calculated. If the CV between the replicates was more than 2.5%, the measurement was repeated. DNA isolated from human embryonic kidney (HEK 293, Gibco, Karlsruhe, Germany) cells was used as control. The PCR data was analyzed with the comparative cycle threshold (Ct) method (2^-ΔΔCt^) [3]. This method measures the relative expression of the telomeric sequence compared to a reference gene (relative telomere length, RTL). All Q-PCR reactions were carried out on a LightCycler (Roche). The age-corrected RTL was calculated by dividing the RTL by age [3].

### Statistical analyses

All data were examined for normality of their frequency distribution using the Kolmogorov-Smirnov test. Non-normally distributed variables were log-transformed prior to further statistical testing. Descriptive statistics provide means (±SD) or medians (10^th^-90^th^ percentiles) for normally and non-normally distributed variables, respectively. Where indicated, quartiles of the entire study cohort were used. We generated quartiles of B12 and age-corrected RTL and the following four groups: LB12LT (low B12 and long telomeres: subjects in the 1^st^ quartile of B12 and in the 4^th^ quartile of age-corrected RTL, n = 174), HB12LT (high B12 and long telomeres: subjects in the 4^th^ quartile of B12 and in the 4^th^ quartile of age-corrected RTL, n= 203), LB12ST (low B12 and short telomeres: subjects in the 1^st^ quartile of B12 and in the 1^st^ quartile of age-corrected RTL, n= 193), and HB12ST (high B12 and short telomeres: subjects in the 4^th^ quartile of B12 and in the 1^st^ quartile of age-corrected RTL, n= 169).

The Kruskal-Wallis test was used to identify differences between multiple groups of continuous variables. The Mann-Whitney-U test was used to compare continuous variables between two independent groups. The chi-square test was used for categorical outcomes. Multiple regression analysis was performed using a backward variable selection. Correlation analyses were performed according to Pearson. The Cox proportional hazard model was used to examine the association between quartiles of B12 and time to death from any cause. Kaplan-Meier curves were calculated to evaluate the cumulative survival during follow-up, according to quartiles of B12 and age-corrected RTL. All tests used were 2-sided and p values <0.05 were considered statistically significant. All statistical analyses were performed using SPSS (Statistical Package for the Social Sciences, version 19.0), and R v3.4.1 (http://www.r-project.org). Kaplan-Meier plots were drawn using the R-package ‘survminer’ (v5.1-1).
